# An anti-cancer derivative of butyric acid (pivalyloxmethyl buterate) and daunorubicin cooperatively prolong survival of mice inoculated with monocytic leukaemia cells.

**DOI:** 10.1038/bjc.1997.151

**Published:** 1997

**Authors:** T. Kasukabe, A. Rephaeli, Y. Honma

**Affiliations:** Department of Chemotherapy, Saitama Cancer Research Institute, Ina, Japan.

## Abstract

A derivative of butyric acid, pivalyloxymethyl butyrate (AN-9), inhibited the proliferation and induced apoptosis of mouse monocytic leukaemia Mm-A cells, although sodium butyrate, but not AN-9, induced differentiation of the cells. AN-9 and DNA-specific antineoplastic agents synergistically inhibited the growth of Mm-A cells, and the simultaneous treatment was required to evoke the maximum growth-inhibitory effect. On the other hand, there was no synergy between butyrate and the drugs, or AN-9 and anti-metabolic agents in inhibiting the growth of the cells, suggesting that the synergistic effect is specific to AN-9 and DNA-reacting agents. AN-9 as a single agent prolonged the survival of mice inoculated with Mm-A cells in a dose-dependent manner. Moreover, administration of AN-9 plus daunorubicin (DNR) markedly prolonged their survival. These results suggest that combination with AN-9 and DNR entails an obvious therapeutic potential.


British Journal of Cancer (1997) 75(6), 850–854
© 1997 Cancer Research Campaign

### An anti-cancer derivative of butyric acid (pivalyloxymethyl butyrate) and daunorubicin cooperatively prolong survival of mice inoculated with monocytic leukaemia cells

**T Kasukabe[1], A Rephaeli[2] and Y Honma[1]**

[1]Department of Chemotherapy, Saitama Cancer Center Research Institute, Ina, Saitama 362, Japan; [2]Ansan Inc., 400 Oyster Point Boulevard, Suite 315, South San Francisco, CA 94080, USA

**Summary** A derivative of butyric acid, pivalyloxymethyl butyrate (AN-9), inhibited the proliferation and induced apoptosis of mouse monocytic leukaemia Mm-A cells, although sodium butyrate, but not AN-9, induced differentiation of the cells. AN-9 and DNA-specific antineoplastic agents synergistically inhibited the growth of Mm-A cells, and the simultaneous treatment was required to evoke the maximum growth-inhibitory effect. On the other hand, there was no synergy between butyrate and the drugs, or AN-9 and anti-metabolic agents in inhibiting the growth of the cells, suggesting that the synergistic effect is specific to AN-9 and DNA-reacting agents. AN-9 as a single agent prolonged the survival of mice inoculated with Mm-A cells in a dose-dependent manner. Moreover, administration of AN-9 plus daunorubicin (DNR) markedly prolonged their survival. These results suggest that combination with AN-9 and DNR entails an obvious therapeutic potential.

**Keywords:** AN-9; butyric acid; daunorubicin; survival; monocytic leukaemia cells

Butyric acid, a non-toxic natural product found in food and present in the digestive system as a byproduct of microbial fermentation, has been shown to be an effective inducer of differentiation in a variety of haematopoietic and non-haematopoietic malignant cells (Prasad, 1980). Clinical trials were conducted with sodium butyrate on haematopoietic malignancies (Novogradsky et al, 1983). It induced a partial and temporary remission in a child with acute myeloid leukaemia (AML), although no clinical activity of butyrate was detected in nine adults with acute leukaemia (Miller et al, 1987). The lack of clinical efficacy may be caused by its rapid metabolism, and to a lesser extent, to its excretion. In order to overcome these disadvantages, a search for novel prodrugs of butyrate, which would impart reduction in clearance rates, was undertaken, and we developed a prodrug, AN-9, which exhibited much greater anti-cancer activity than butyrate in vitro and in vivo (Nudelman et al, 1992; Rephaeli et al, 1991).

Some DNA-specific antileukaemic drugs induced differentiation of leukaemia cells in conjunction with non-toxic differentiation inducers (Okabe et al, 1979; Honma et al, 1986). The two components interact synergistically and differentiation can, therefore, be induced at concentrations at which neither drug nor inducer, administered alone, are capable of effecting differentiation to a significant extent. Since differentiation is accompanied by the loss of proliferative capacity, a chemodifferentiation approach, based on drug–non-toxic inducer interactions, holds therapeutic promise.

*Received 10 June 1996*
*Revised 17 September 1996*
*Accepted 20 September 1996*

*Correspondence to:* T Kasukabe

The remission rate of acute monocytic leukaemia (AMMOL) is lower than that of the other types of AML, and even when remission is achieved by treatment with conventional cytotoxic antileukaemic drugs, the median duration of remission is only about 6 months (Fenaux et al, 1990). Therefore, it is important to develop new strategies for control of AMMOL that are refractory to conventional cytotoxic antileukaemic drugs. The mouse monocytic leukaemia Mm-A cells are leukaemogenic to syngeneic mice and can phagocyoise sensitized sheep erythrocytes, produce lysozyme and adhere to culture dishes (Kasukabe et al, 1984). The experimental system is provided to study therapeutic strategies for monocytic leukaemia. Butyrate was the most effective agent for increasing lysozyme production by Mm-A cells, and it also stimulated other differentiation-associated functions (Kasukabe et al, 1985). In the primary culture of AML cells, butyrate was effective in inducing morphological and functional differentiation of leukaemia cells in some cases obtained from AMMOL patients (Honma et al, 1983; Rephaeli et al, 1994). These results suggest that butyrate and/or its derivatives may be useful in the therapy of AMMOL. In the present investigation, we have compared the effect of butyrate and its derivative, AN-9, on the proliferation of Mm-A cells in conjunction with some antileukaemic drugs, and examined the therapeutic effect of AN-9 on mice inoculated with Mm-A cells.

### MATERIALS AND METHODS

### Materials

AN-9 was prepared from butyric acid and chloromethyl pivalate in the presence of triethyl amine (Nudelman et al, 1992) and

**Figure 1** Effects of sodium butyrate and AN-9 on the growth and lysozyme production of Mm-A cells. Cells were cultured with various concentrations of sodium *n*-butyrate (open symbols) or AN-9 (closed symbols) for 4 days dissolved in dimethyl sulphoxide. Daunorubicin (DNR), doxorubicin, actinomycin D, 3-[4,5-dimethylthiazol-2-yl]-2,5-diphenyltetrazolium (MTT) and nitroblue tetrazolium (NBT) were purchased from Sigma (St Louis, MO, USA).

### Cells and cell culture

Mouse monocytic leukaemia Mm-A cells were cultured in Eagle's minimum essential medium with 10% heat-inactivated calf serum at 37°C in a humidified atmosphere of 5% carbon dioxide in air (Kasukabe et al, 1984). Human myeloid leukaemia cell lines were cultured in RPMI-1640 medium with 10% heat-inactivated fetal bovine serum (Makishima et al, 1991; Okabe-Kado et al, 1991).

### Assay of cell growth, differentiation-associated properties and apoptosis

Suspensions of cells ($5 \times 10^4$ cells per ml) were cultured with various concentrations of test chemicals in 24-well culture dishes for 4–6 days. Then, cell numbers were counted in a model ZM Coulter counter (Coulter Electronics, Luton, UK). Viable cells were examined by MTT assay, as described previously (Kanatani et al, 1993).

Lysozyme activity and NBT reduction were assayed as reported previously (Kanatani et al, 1993). Morphological changes were examined in cell smears stained with May–Gruenwald–Giemsa solution.

### Animals

Inbred SL strain mice were maintained as previously reported and female mice were used at 8–10 weeks old (Honma et al, 1978). The mice were inoculated i.p. with $6 \times 10^6$ Mm-A cells per animal.

### Administration of AN-9 and DNR

For the assay of in vivo antileukaemic effect of AN-9, it was prepared in 20% Intralipid (Green Cross, Osaka, Japan). DNR was dissolved in phosphate-buffered saline. The mice were given i.p.

**Table 1** Growth-inhibitory effect of butyrate and AN-9 on several myeloid leukaemia cells

| Cells | Growth inhibition ($IC_{50}$, μM) | |
|---|---|---|
| | Butyrate | AN-9 |
| Mouse | | |
| Mm-A (monocytoid) | 253 ± 18 | 105 ± 11 |
| Human | | |
| HL-60 (promyelocytic) | 691 ± 54 | 51 ± 7 |
| U937 (monocytoid) | 943 ± 87 | 148 ± 18 |
| THP-1 (monocytoid) | 741 ± 69 | 140 ± 16 |
| HEL/S (monocytoid) | 869 ± 78 | 119 ± 12 |

Cells were cultured with various concentrations of sodium butyrate or AN-9 for 4 days, and the concentration of the drug required for 50% inhibition of cell growth ($IC_{50}$) was examined. Values are mean ± s.d.

injections of 0.2 ml of each solution at doses of 2.5 mg per mouse (109 mg $kg^{-1}$) of AN-9 and 3 μg per mouse (130 μg $kg^{-1}$) of DNR. The first dose was administered 1 day after the leukaemia cell inoculation.

### Analysis of combined drug effects

Isobologram analysis was the basis for analysing combined drug effects with leukaemia cells (Berenbaum, 1989). Dose effects were determined for each compound and for one compound in fixed concentrations of another one. The interaction of two compounds was quantified by determining a combination index (CI) value according to the classic isobologram equation. This analysis generates the combination effect as: summation (additivity or zero interaction) is indicated when CI = 1; synergism is indicated when CI < 1; antagonism is indicated when CI > 1.

### RESULTS

### Combined effect of AN-9 and DNR on proliferation of Mm-A cells

AN-9 inhibited the growth of Mm-A cells at lower concentrations than sodium *n*-butyrate (Figure 1). Butyrate effectively enhanced lysozyme production of Mm-A cells, as described previously (Kasukabe et al, 1985), whereas AN-9 did not affect lysozyme production of the cells, even in a high concentration. Nor did AN-9 induce differentiation as assessed by other markers, such as NBT reduction (data not shown), although AN-9 induced NBT reduction of human myeloid leukaemia HL-60 cells (Rephaeli et al, 1991). The AN-9-treated Mm-A cells became non-adherent, and morphological examination revealed shrivelled cells, chromatin condensation, nuclear fragmentation and cytoplasmic blebbing in the culture of Mm-A cells treated with AN-9 for 7 days. These results indicate that AN-9 induced apoptosis rather than differentiation of Mm-A cells. Next, we examined the growth-inhibitory effect of AN-9 on various leukaemia cell lines. Table 1 shows that HL-60 cells were the most sensitive to the growth-inhibitory activity of AN-9, while Mm-A cells were more sensitive to butyrate than HL-60 cells. With respect to growth inhibition by AN-9, Mm-A cells were more sensitive than human monocytoid leukaemia U937, HEL/S and THP-1 cells. Lysozyme production of the monocytoid leukaemia cells was induced by butyrate, but not by AN-9 (data not shown). These results suggest that the effect

© Cancer Research Campaign 1997 

**Figure 2** Combined effect of sodium butyrate or AN-9 with DNR on the growth of Mm-A cells. Cells were cultured with various concentrations of DNR in the presence of 0 (■), 30 (●), 45 (▲) or 60 (◆) µM AN-9 for 4 days (**A**). Cells were cultured with DNR in the presence of 0 (■), 0.1 (●), 0.2 (▲) or 0.3 (◆) mM sodium butyrate for 4 days (**B**)

**Table 2** Synergy between AN-9 and various anti-cancer drugs in growth inhibition of Mm-A cells

| Drug | $IC_{50}$(M) – AN-9 | $IC_{50}$(M) + AN-9 | CI[a] |
|---|---|---|---|
| DNR | $1.06 \times 10^{-7}$ | $0.44 \times 10^{-7}$ | 0.49 |
| Doxorubicin | $6.79 \times 10^{-8}$ | $3.45 \times 10^{-8}$ | 0.53 |
| Actinomycin D | $1.27 \times 10^{-9}$ | $0.48 \times 10^{-9}$ | 0.48 |
| Cytosine arabinoside | $2.07 \times 10^{-7}$ | $2.32 \times 10^{-7}$ | 1.03 |
| Methotrexate | $1.10 \times 10^{-7}$ | $1.16 \times 10^{-7}$ | 1.06 |
| 6-Mercaptopurine | $6.58 \times 10^{-8}$ | $6.56 \times 10^{-8}$ | 0.99 |
| 5-Fluorouracil | $7.69 \times 10^{-7}$ | $7.81 \times 10^{-7}$ | 1.08 |
| Etoposide | $6.38 \times 10^{-7}$ | $6.42 \times 10^{-7}$ | 1.04 |

Cells were cultured with various concentrations of antineoplastic agents in the presence or absence of 30 µM AN-9. [a]Combination index at $IC_{50}$. CI = 1 indicates summation (additive or zero interaction), CI > 1 antagonism, CI < 1 synergism.

**Table 3** Effect of sequence of application of various combinations of DNR and AN-9 on the proliferation of Mm-A cells

| Regimen | Additions on Days 1–3 | Additions on Days 4–6 | Cell number on day 6 $\times 10^5$ ml$^{-1}$ | Cell number on day 6 Percentage of control |
|---|---|---|---|---|
| 1 | None | None | 14.2 ± 1.6 | 100 |
| 2 | AN-9 | None | 14.0 ± 1.8 | 99 |
| 3 | None | AN-9 | 14.3 ± 1.7 | 100 |
| 4 | AN-9 | AN-9 | 12.4 ± 1.3 | 87 |
| 5 | DNR | None | 11.2 ± 1.1 | 79 |
| 6 | None | DNR | 12.5 ± 1.4 | 87 |
| 7 | DNR | DNR | 8.8 ± 0.9 | 62 |
| 8 | DNR | AN-9 | 10.0 ± 1.2 | 70 |
| 9 | AN-9 | DNR | 12.6 ± 1.5 | 89 |
| 10 | AN-9 + DNR | None | 3.3 ± 0.5 | 23 |
| 11 | None | AN-9 + DNR | 9.4 ± 1.1 | 66 |

Cells were cultured for 3 days in the absence or presence of 20 ng ml$^{-1}$ DNR or 46.5 µM AN-9. On day 3, the cultures were washed with fresh medium and reincubated in the presence or absence of the same concentration of DNR or AN-9.

of AN-9 is different from that of butyrate in monocytoid leukaemia cells.

When Mm-A cells were cultured with 20 ng ml$^{-1}$ DNR for 4 days, the growth was hardly affected. In the presence of low concentrations of AN-9, DNR-induced growth inhibition was evident, whereas butyrate did not affect DNR-induced growth inhibition of Mm-A cells (Figure 2). These synergistic effects between AN-9 and DNR were also observed in 3-day treatment (data not shown). Next, we examined the combined effect of various anti-cancer drugs with AN-9 on the growth of Mm-A cells. Actinomycin D and doxorubicin were also effective in the synergistic inhibition of growth in the presence of AN-9, whereas no synergistic effect with AN-9 was observed in combination with cytosine arabinoside, 5-fluorouracil, methotrexate, etoposide or 6-mercaptopurine (Table 2). Expression of differentiation-associated phenotypes of Mm-A cells was not significantly affected by the combined treatment with AN-9 and DNR (data not shown). Treatment of the cells with AN-9 for 3 days caused no significant decrease in cell numbers (Table 3, regimens 2 and 3). When AN-9 was added for an additional 3 days (regimen 4), the cell number decreased very slightly. At the concentration of AN-9 used, cell

© Cancer Research Campaign 1997

Table 4 Effect of AN-9 on survival times of mice inoculated with Mm-A cells

| Experiment | Dose (mg per mouse) | No.of mice | Survival (days ± s.d.) | T/C[a] |
|---|---|---|---|---|
| I | 0 | 10 | 22.3 ± 2.8 | |
| | 3 | 5 | 36.8 ± 6.5* | 165 |
| II | 0 | 20 | 21.7 ± 1.6 | |
| | 1 | 5 | 21.6 ± 2.4 | 100 |
| | 2.5 | 10 | 31.7 ± 3.6** | 146 |
| III | 0 | 10 | 22.6 ± 2.2 | |
| | 2.5 | 5 | 30.9 ± 5.9* | 137 |
| | 5 | 5 | 40.4 ± 7.1** | 179 |

SL mice were inoculated with 6 × $10^6$ Mm-A cells and treated with AN-9 every other day. [a]Ratio of survival days of treated mice to those of solvent control. *$P < 0.01$, **$P < 0.001$, using Student's *t*-test. In experiment I, 50% propylene glycol was used as solvent instead of 20% Intralipid.

**Figure 3** Effects of AN-9 and DNR on the survival of SL mice inoculated with monocytic leukaemia Mm-A cells. SL female mice were inoculated i.p. with 6 × $10^6$ Mm-A cells. One day after the inoculation, the mice were treated with solvent alone (—), 2.5 mg of AN-9 (— —), 3 μg of DNR (- - - -) or AN-9 plus DNR (······) every other day. Administration of DNR was done on days 1, 3, 5 and 7, and AN-9 was administered 11 times. Ten mice were used in a group of 'solvent alone' or 'AN-9 plus DNR', and five mice were used in a group of 'AN-9' or 'DNR'. The survival time of mice treated with AN-9 plus DNR was prolonged more than that of mice treated with AN-9 alone ($P < 0.05$, log-rank test)

viability was in excess of 95% (data not shown). When the cells were cultured with DNR for 3 days (regimens 5 and 6), the growth decreased slightly, whereas the administration of a second dose of DNR on day 3 (regimen 7) caused significant inhibition of cell growth. Treatment of the cells for 3 days with DNR, followed by a 3-day exposure to AN-9 (regimen 8) resulted in a more pronounced decrease in cell number than the administration of AN-9 before DNR (regimen 9). When the drug and AN-9 were administered simultaneously (regimen 10), approximately 77% growth inhibition was observed. When the DNR–AN-9 combination was applied for the second 3 days (regimen 11), the growth inhibition was similar to that in regimen 7. These results indicate that the simultaneous treatment is the most effective in combinations of DNR and AN-9.

### Effect of AN-9 on the survival of mice inoculated with Mm-A cells

After inoculation of 6 × $10^6$ Mm-A cells, all the mice died of leukaemia within 30 days. At necropsy, the abdomen of mice contained a large amount of ascites with numerous Mm-A cells, and many small tumour nodules were scattered on the liver surface, mesentery and peritoneum. In some cases, splenomegaly and hepatic colonization of the leukaemia cells were observed, as in AMMOL patients (Shaw, 1978). To test the effect of AN-9 alone on the leukaemogenicity of the monocytic leukaemia cells, we administered AN-9 i.p. to syngeneic SL mice inoculated with Mm-A cells. Since AN-9 possesses low toxicity ($LD_{50}$ in mice is 1.36 g $kg^{-1}$) (Rephaeli et al, 1991), no appreciable side-effect was observed in our experiments. AN-9 significantly prolonged the mean survival time of mice inoculated with Mm-A cells, and the therapeutic effect of AN-9 was dose dependent (Table 4).

Mice inoculated with the monocytic leukaemia cells were injected with DNR, a representative antileukaemic agent, the first injection being given 1 day after tumour challenge. This treatment significantly prolonged the survival of the mice (Figure 3). Although the drug was fairly toxic to SL mice when injected with a higher dose of DNR, all the mice treated with 3 μg of DNR survived for a long time (more than 3 months), and no appreciable side-effects were observed.

Next, we examined the therapeutic effect of the combined treatment with AN-9 and DNR. The median survival time of mice inoculated with Mm-A cells was 31 days on treatment with AN-9 only, but 47 days on treatment with 2.5 mg of AN-9 and 3 μg of DNR ($P < 0.05$, log-rank test)(Figure 3). The in vivo finding on the prolongation of survival times is compatible with that on the synergistic effect of AN-9 and DNR on the growth of the cells.

### DISCUSSION

AN-9 is a butyric acid prodrug, but its effect on the growth and differentiation of monocytic leukaemia cells is different from that of butyrate. Butyrate and AN-9 modulated the expression of the early regulating genes, *c-myc* and *c-jun*, but AN-9 elicited this effect at least 100 times faster than butyrate (Rabizadeh et al, 1993). This may be caused by a faster rate of intracellular penetration by the lipophilic AN-9 and/or a slower rate of metabolic degradation. The in vitro and in vivo antileukaemic activity of AN-9 may be attributable to its pharmacokinetic properties.

With respect to the growth-inhibitory effect, there was synergy between AN-9 (but not butyrate) and DNA-specific antineoplastic agents, such as DNR, doxorubicin and actinomycin D, but there was no synergy between AN-9 and other anti-cancer drugs, such as etoposide, methotrexate, 6-mercaptopurine and cytosine arabinoside. These results suggest that the synergistic effect was specific to DNA-reacting agents in conjunction with AN-9. Simultaneous treatment with AN-9 and DNR was required to evoke the maximum effect in inhibiting the growth of Mm-A cells. Both AN-9 and butyrate caused a transient hyperacetylation of histones, and AN-9 induced at a concentration one order of magnitude lower than butyrate (Aviram et al, 1994). The kinetics of AN-9-induced histone acetylation were faster than those of butyrate. The histone hyperacetylation loosened the chromatin structure (Lee et al, 1993), and this may improve the accessibility of DNR to nucleosomal DNA. A reversible increase of histone acetylation by AN-9 and the requirement of simultaneous treatment with AN-9 and DNR are compatible with this view. Alternatively, there may be a reasonable mechanism in which AN-9 has synergy with intercalating agents, such as DNR, doxorubicin and actinomycin D, but not other classes of antineoplastic agents. However, the mechanism of interaction between DNA-specific antineoplastic agents and AN-9 remains to be elucidated.

AMMOL is more refractory to conventional cytotoxic antileukaemic drugs than other subtypes of AML (Fenaux et al, 1990).

© Cancer Research Campaign 1997

Mouse leukaemia Mm-A cells exhibit several monocyte-associated phenotypes and induce monocytic leukaemia in syngeneic SL mice (Kasukabe et al, 1984). The leukaemic mouse inoculated with Mm-A cells is a good experimental model of AMMOL. AN-9 possessed low toxicity and displayed anti-tumour activity in B16 melanoma and Lewis lung carcinoma models (Rephaeli et al, 1991; Nudelman et al, 1992), as well as the Mm-A monocytic leukaemia model. Combination therapy with DNR and AN-9 is definitely more effective than therapy with DNR alone in the leukaemic mice inoculated with Mm-A cells. Human monocytoid leukaemia cell lines had similar sensitivity to AN-9 in inhibiting cell growth (Table 1). Since synergy requires the use of only minimally toxic drug concentrations, the approach is preferred over maximally tolerated dose regimens that are highly toxic to the host. With respect to AMMOL, more effective therapy with fewer side-effects might be achieved with a combination of DNR and AN-9. Further experiments on the synergistic effects of AN-9 and DNR should result in the development of new strategies of AMMOL therapy.

### ACKNOWLEDGEMENT

This work was supported in part by a Grant-in-Aid for Cancer Research from the Ministry of Education, Science and Culture of Japan.

### REFERENCES

Aviram A, Zimrah Y, Shaklai M, Nudelman A and Rephaeli A (1994) Comparison between the effect of butyric acid and its prodrug pivalyloxymethylbutyrate on histones hyperacetylation in an HL-60 leukemic cell line. *Int J Cancer* **56**: 906–909

Berenbaum M C (1989) What is synergy? *Pharmacol Rev* **41**: 93–141

Fenaux P, Vanhaesbroucke C, Estienne M H, Homme C P, Pagniez D, Facon T, Millot F and Bauters F (1990) Acute monocytic leukaemia in adults: treatment and prognosis in 99 cases. *Br J Haematol* **75**: 41–48

Honma Y, Kasukabe T and Hozumi M (1978) Relationship between leukemogenicity and *in vivo* inducibility of normal differentiation in mouse myeloid leukemia cells. *J Natl Cancer Inst* **61**: 837–841

Honma Y, Fujita Y, Kasukabe T, Hozumi M, Sampi K, Sakurai M, Tsushima S and Nomura H (1983) Induction of differentiation of human acute non-lymphocytic leukemia cells in primary culture by inducers of differentiation of human myeloid leukemia cell line HL-60. *Eur J Cancer Clin Oncol* **19**: 251–261

Honma Y, Honma C and Bloch A (1986) Mechanism of interaction between antineoplastic agents and natural differentiation factors in the induction of human leukemic cell maturation. *Cancer Res* **46**: 6311–6315

Kanatani Y, Kasukabe T, Hozumi M, Motoyoshi K, Nagata N and Honma Y (1993) Genistein exhibits preferential cytotoxicity to a leukemogenic variant but induces differentiation of a non-leukemogenic variant of the mouse monocytic leukemia Mm cell line. *Leuk Res* **17**: 847–853

Kasukabe T, Honma Y and Hozumi M (1984) Selection of mouse macrophage-like sublines that differ in leukemogenic potential and characterization. *J Cell Physiol* **118**: 105–112

Kasukabe T, Honma Y and Hozumi M (1985) Induction of differentiation of cultured mouse monocytic leukemia cells (Mm-A) by inducers different from those of parent myeloblastic leukemia cells (M1). *Jpn J Cancer Res* **76**: 1056–1063

Lee D, Hayes J, Pruss D and Wolffe A (1993) A positive role for histone acetylation in transcription factor access to nucleosomal DNA. *Cell* **72**: 73–84

Makishima M, Honma Y, Hozumi M, Sampi K, Hattori M and Motoyoshi K (1991) Induction of differentiation of human leukemia cells by inhibitors of myosin light chain kinase. *FEBS Lett* **287**: 175–177

Miller A A, Kurschel E, Osieka R and Schmidt C (1987) Clinical pharmacology of sodium butyrate in patients with acute leukemia. *Eur J Cancer Clin Oncol* **23**: 1283–1287

Novogradsky A, Dvir A, Ravid A, Shkolnik T, Stenzel K H, Rubin A L and Zaizov R (1983) Effect of polar organic compounds on leukemic cells. *Cancer* **51**: 9–14

Nudelman A, Ruse M, Aviram A, Rabizadeh E, Shaklai M, Zimrah Y and Rephaeli A (1992) Novel anticancer prodrugs of butyric acid. *J Med Chem* **35**: 687–694

Okabe J, Honma Y and Hozumi M (1979) Actinomycin D restores *in vivo* sensitivity to differentiation induction of non-differentiating mouse myeloid leukemia cells. *Int J Cancer* **24**: 87–91

Okabe-Kado J, Honma Y, Hayashi M and Hozumi M (1991) Effects of transforming growth factorβ and activin A on vitamin $D_3$-induced monocytic differentiation of myeloid leukemia cells. *Anticancer Res* **11**: 181–186

Prasad K N (1980) Butyric acid: a small fatty acid with diverse biological functions. *Life Sci* **27**: 1351–1358

Rabizadeh E, Shaklai M, Nudelman A, Eisenbach L and Rephaeli A (1993) Rapid alteration of c-*myc* and c-*jun* expression in leukemic cells induced to differentiate by a butyric acid prodrug. *FEBS Lett* **328**: 225–229

Rephaeli A, Nordenberg J, Aviram A, Rabizadeh E, Zimra Y, Nudelman A, Novogrodsky A and Shaklai M (1994) Butyrate-induced differentiation in leukemic myeloid cells: *in vitro* and *in vivo* studies. *Int J Oncol* **4**: 1387–1391

Rephaeli A, Rabizadeh E, Aviram A, Shaklai M, Ruse M and Nudelman A (1991) Derivatives of butyric acid as potential anti-neoplastic agents. *Int J Cancer* **49**: 66–72

Shaw M T (1978) The distinctive features of acute monocytic leukemia. *Am J Hematol* **4**: 97–103

© Cancer Research Campaign 1997


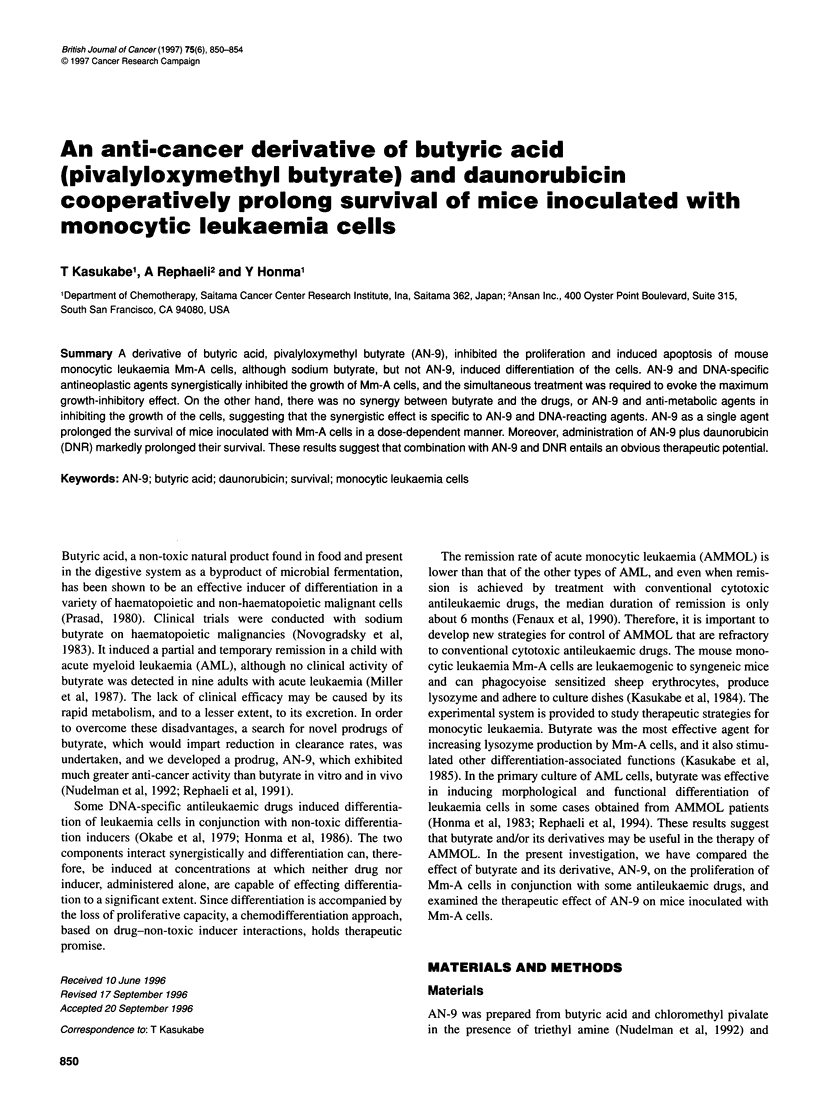

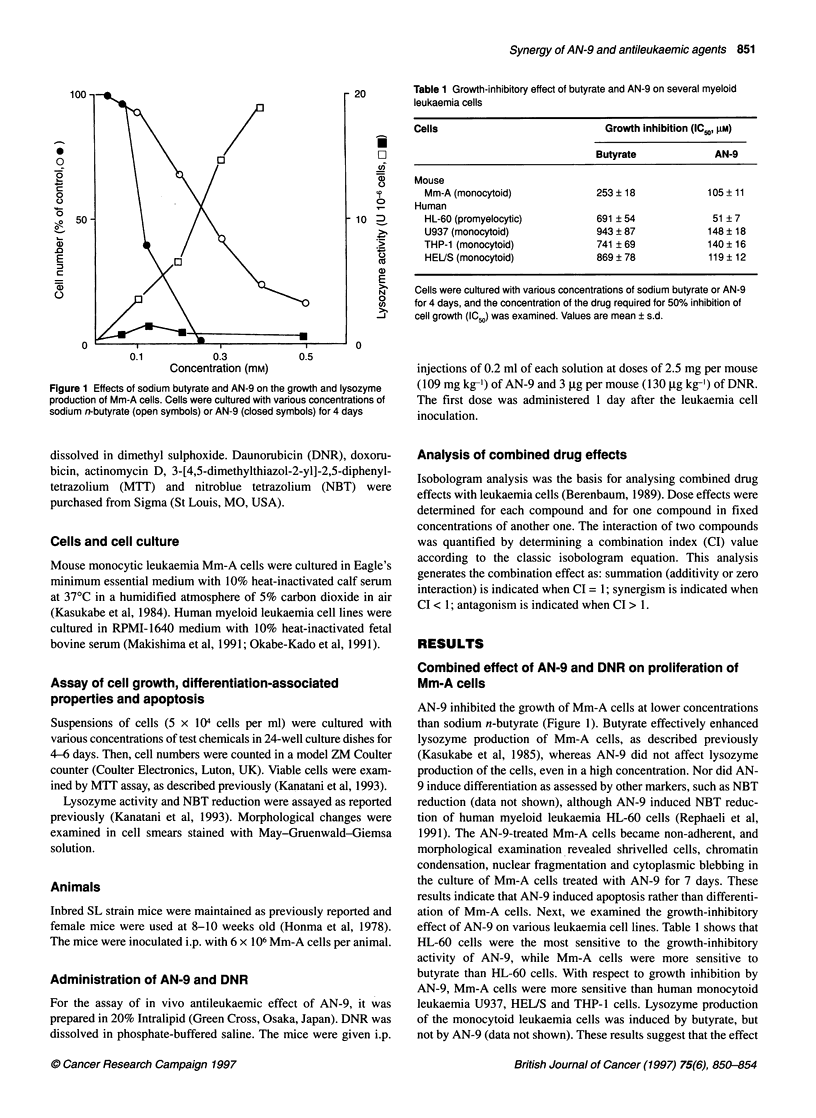

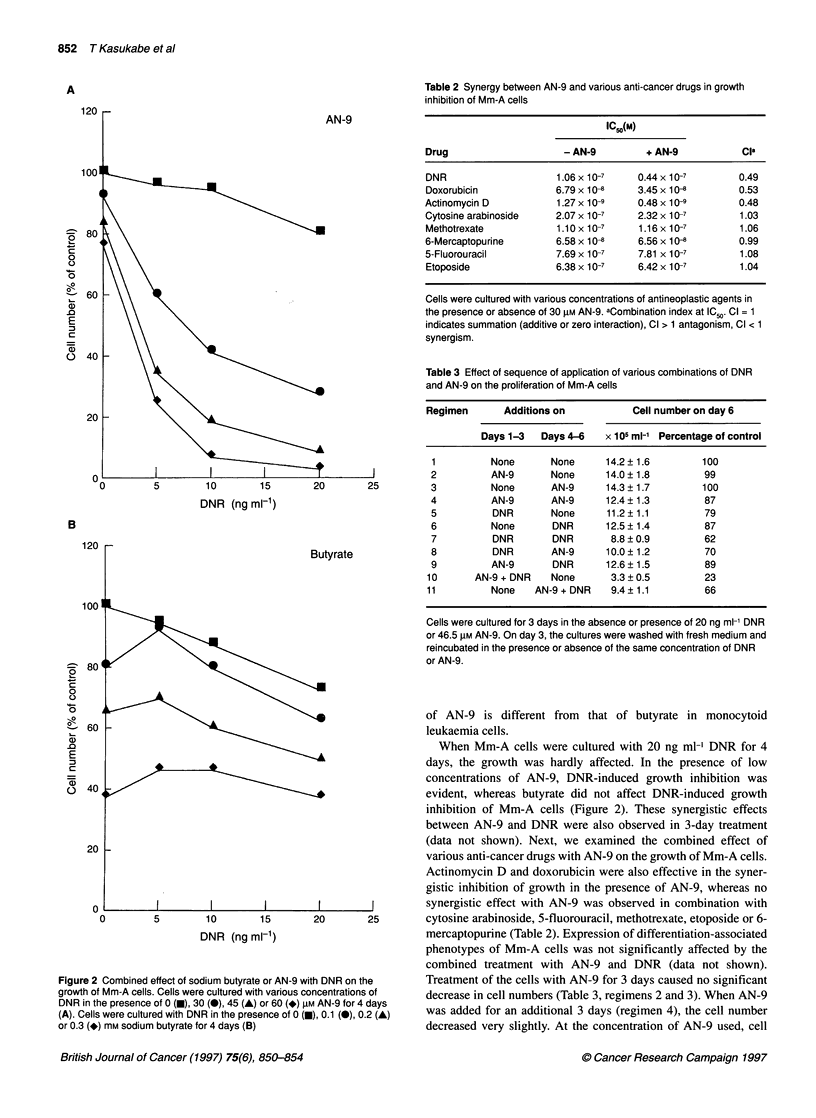

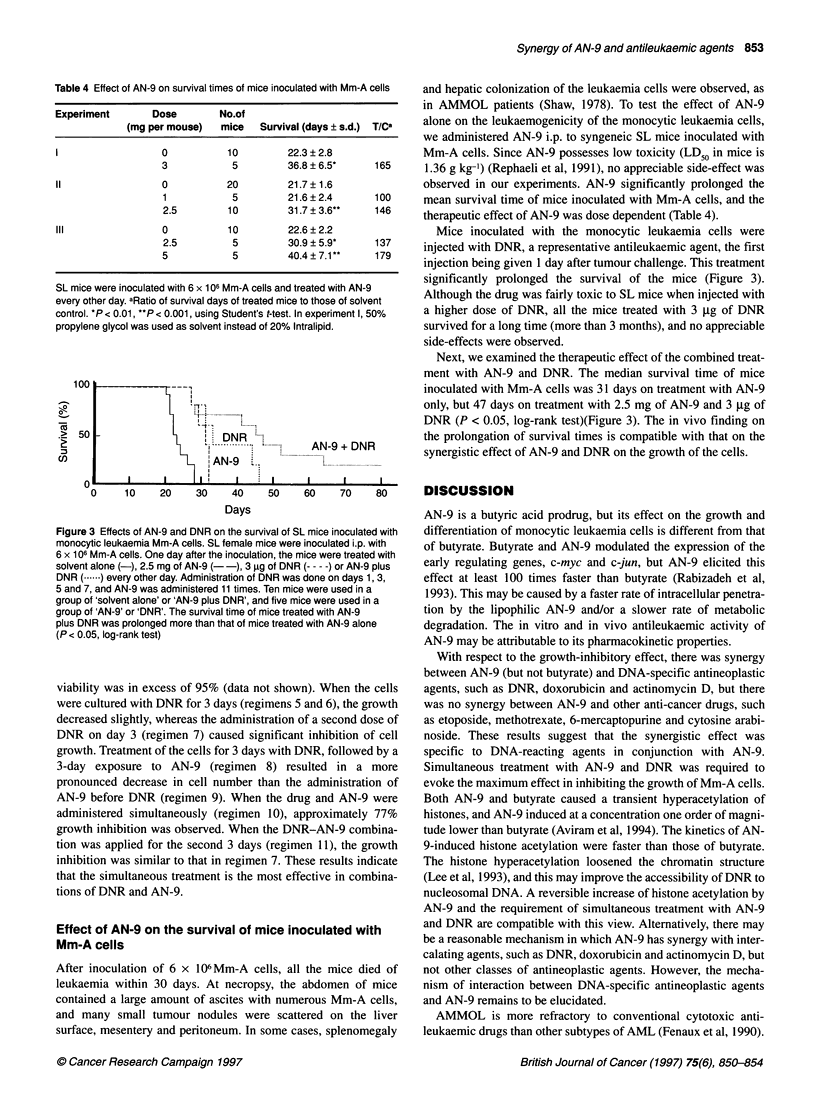

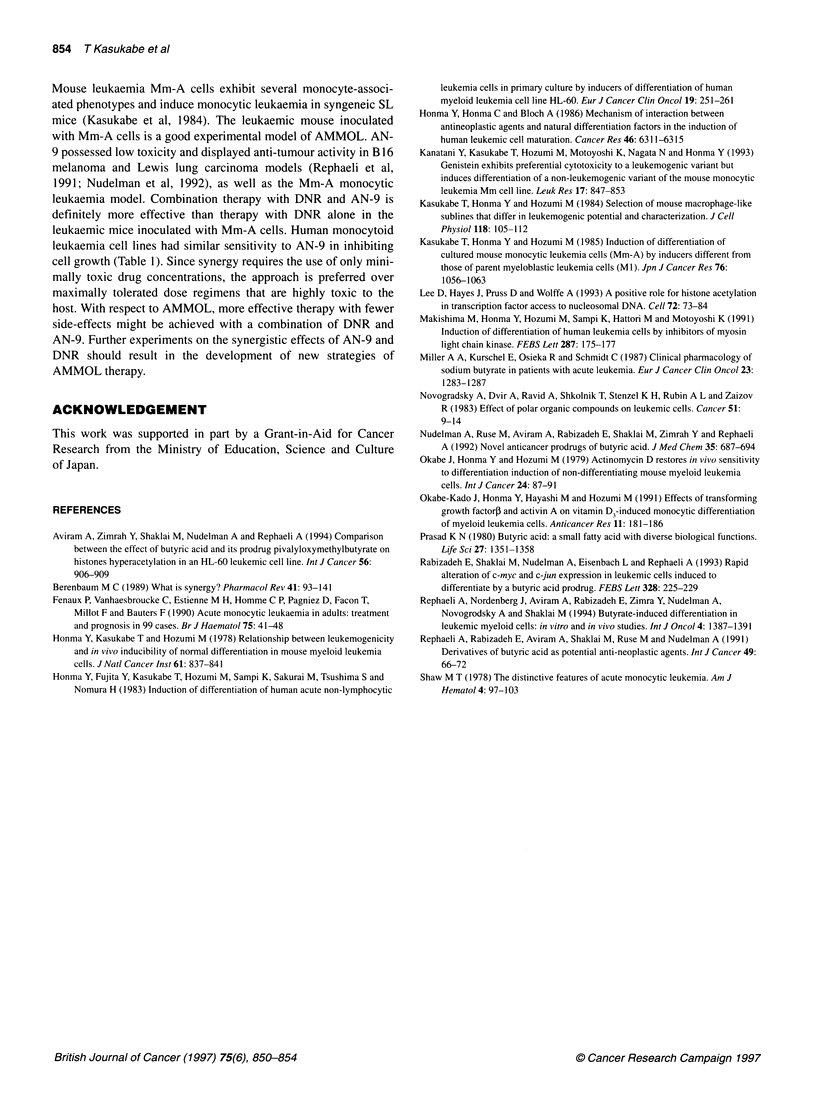


## References

[OCR_00572] Aviram A., Zimrah Y., Shaklai M., Nudelman A., Rephaeli A. (1994). Comparison between the effect of butyric acid and its prodrug pivaloyloxymethylbutyrate on histones hyperacetylation in an HL-60 leukemic cell line.. Int J Cancer.

[OCR_00578] Berenbaum M. C. (1989). What is synergy?. Pharmacol Rev.

[OCR_00582] Fenaux P., Vanhaesbroucke C., Estienne M. H., Preud'homme C., Pagniez D., Facon T., Millot F., Bauters F. (1990). Acute monocytic leukaemia in adults: treatment and prognosis in 99 cases.. Br J Haematol.

[OCR_00590] Honma Y., Fujita Y., Kasukabe T., Hozumi M., Sampi K., Sakurai M., Tsushima S., Nomura H. (1983). Induction of differentiation of human acute non-lymphocytic leukemia cells in primary culture by inducers of differentiation of human myeloid leukemia cell line HL-60.. Eur J Cancer Clin Oncol.

[OCR_00596] Honma Y., Honma C., Bloch A. (1986). Mechanism of interaction between antineoplastic agents and natural differentiation factors in the induction of human leukemic cell maturation.. Cancer Res.

[OCR_00585] Honma Y., Kasukabe T., Hozumi M. (1978). Relationship between leukemogenicity and in vivo inducibility of normal differentiation in mouse myeloid leukemia cells.. J Natl Cancer Inst.

[OCR_00601] Kanatani Y., Kasukabe T., Hozumi M., Motoyoshi K., Nagata N., Honma Y. (1993). Genistein exhibits preferential cytotoxicity to a leukemogenic variant but induces differentiation of a non-leukemogenic variant of the mouse monocytic leukemia Mm cell line.. Leuk Res.

[OCR_00613] Kasukabe T., Honma Y., Hozumi M. (1985). Induction of differentiation of cultured mouse monocytic leukemia cells (Mm-A) by inducers different from those of parent myeloblastic leukemia cells (M1).. Jpn J Cancer Res.

[OCR_00608] Kasukabe T., Honma Y., Hozumi M. (1984). Selection of mouse macrophage-like sublines that differ in leukemogenic potential and characterization.. J Cell Physiol.

[OCR_00619] Lee D. Y., Hayes J. J., Pruss D., Wolffe A. P. (1993). A positive role for histone acetylation in transcription factor access to nucleosomal DNA.. Cell.

[OCR_00623] Makishima M., Honma Y., Hozumi M., Sampi K., Hattori M., Motoyoshi K. (1991). Induction of differentiation of human leukemia cells by inhibitors of myosin light chain kinase.. FEBS Lett.

[OCR_00628] Miller A. A., Kurschel E., Osieka R., Schmidt C. G. (1987). Clinical pharmacology of sodium butyrate in patients with acute leukemia.. Eur J Cancer Clin Oncol.

[OCR_00633] Novogrodsky A., Dvir A., Ravid A., Shkolnik T., Stenzel K. H., Rubin A. L., Zaizov R. (1983). Effect of polar organic compounds on leukemic cells. Butyrate-induced partial remission of acute myelogenous leukemia in a child.. Cancer.

[OCR_00638] Nudelman A., Ruse M., Aviram A., Rabizadeh E., Shaklai M., Zimrah Y., Rephaeli A. (1992). Novel anticancer prodrugs of butyric acid. 2.. J Med Chem.

[OCR_00646] Okabe-Kado J., Honma Y., Hayashi M., Hozumi M. (1991). Effects of transforming growth factor-beta and activin A on vitamin D3-induced monocytic differentiation of myeloid leukemia cells.. Anticancer Res.

[OCR_00641] Okabe J., Honma Y., Hayashi M., Hozumi M. (1979). Actinomycin D restores in vivo sensitivity to differentiation induction of non-differentiating mouse myeloid leukemia cells.. Int J Cancer.

[OCR_00651] Prasad K. N. (1980). Butyric acid: a small fatty acid with diverse biological functions.. Life Sci.

[OCR_00655] Rabizadeh E., Shaklai M., Nudelman A., Eisenbach L., Rephaeli A. (1993). Rapid alteration of c-myc and c-jun expression in leukemic cells induced to differentiate by a butyric acid prodrug.. FEBS Lett.

[OCR_00665] Rephaeli A., Rabizadeh E., Aviram A., Shaklai M., Ruse M., Nudelman A. (1991). Derivatives of butyric acid as potential anti-neoplastic agents.. Int J Cancer.

[OCR_00670] Shaw M. T. (1978). The distinctive features of acute monocytic leukemia.. Am J Hematol.

